# Expression of Toll-like receptors in the cerebellum during pathogenesis of prion disease

**DOI:** 10.3389/fnbeh.2024.1341901

**Published:** 2024-04-18

**Authors:** Xiangyu Liao, Wufei Zhu, Xingyu Liao, Wensen Liu, Yiwei Hou, Jiayu Wan

**Affiliations:** ^1^Department of Oncology, Yichang Central People's Hospital, The First College of Clinical Medical Science, China Three Gorges University, Yichang, China; ^2^Department of Endocrinology, Yichang Central People's Hospital, The First College of Clinical Medical Science, China Three Gorges University, Yichang, China; ^3^Computational Bioscience Research Center (CBRC), Computer, Electrical and Mathematical Sciences and Engineering Division, King Abdullah University of Science and Technology (KAUST), Jeddah, Saudi Arabia; ^4^Changchun Veterinary Research Institute, Chinese Academy of Agricultural Sciences, Changchun, China

**Keywords:** prion disease, Toll-like receptor, *PrP^Sc^*, innate immune, central nervous system

## Abstract

Prion diseases, such as scrapie, entail the accumulation of disease-specific prion protein (*PrP*^*Sc*^) within the brain. Toll-like receptors (TLRs) are crucial components of the pattern recognition system. They recognize pathogen-associated molecular patterns (PAMPs) and play a central role in orchestrating host innate immune responses. The expression levels of Toll-like receptors (TLRs) in the central nervous system (CNS) were not well-defined. To establish a model of prion diseases in BALB/C mice, the 22L strain was employed. The features of the 22L strain were analyzed, and the cerebellum exhibited severe pathological changes. TLR1-13 levels in the cerebellum were measured using quantitative polymerase chain reaction (qPCR) at time points of 60, 90, 120, and the final end point (145 days post-infection). During the pathogenesis, the expression levels of Toll-like receptors (TLRs) 1, 2, 7, 8, and 9 increased in a time-dependent manner. This trend mirrored the expression patterns of *PrP*^*Sc*^ (the pathological isoform of the prion protein) and glial fibrillary acidic protein. Notably, at the end point, TLR1-13 levels were significantly elevated. Protein level of TLR7 and TLR9 showed increasing at the end point of the 22L-infected mice. A deeper understanding of the increased Toll-like receptors (TLRs) in prion diseases could shed light on their role in initiating immune responses at various stages during pathogenesis. This insight is particularly relevant when considering TLRs as potential therapeutic targets for prion diseases.

## 1 Introduction

Transmissible spongiform encephalopathies (TSEs), also known as prion diseases, are a group of rare progressive neurodegenerative disorders that occur in both humans and animals. These diseases encompass a range of specific conditions, including: Creutzfeldt–Jakob disease (CJD) (Hermann et al., 2021), Fatal familial insomnia (FFI) (Chu et al., 2022), Gerstmann–Straussler–Scheinker (GSS) syndrome (Ahn et al., 2022), Kuru (observed in humans) (Kothekar and Chaudhary, 2024), Scrapie (affecting sheep and goats) (Acín and Bolea, 2021), Bovine spongiform encephalopathy (BSE or “mad cow disease” in cattle) (Balamuralidhara, 2023), Chronic wasting disease (CWD) in cervids (deer, elk, and related species) (Silva, 2022), Transmissible mink encephalopathy (TME) in mink (Moore et al., 2019). These conditions share common features, such as long incubation periods, spongiform changes in brain tissue, and a lack of inflammatory response. The underlying cause of TSEs is believed to be abnormal prion proteins, which induce misfolding of normal cellular proteins. Unfortunately, prion diseases are invariably fatal.

The neuropathology of prion diseases is marked by the presence and aggregation of a misfolded, insoluble, and protease-resistant form (*PrP*^*Sc*^) of the cellular prion protein (PrPc) within the central nervous system. This accumulation of *PrP*^*Sc*^ contributes to characteristic neurological features, including spongiform degeneration, gliosis, and neuronal cell death (Ritchie and Ironside, 2017). Toll-like receptors (TLRs) are an essential family of receptors expressed by innate immune cells. They play a crucial role in activating the immune response against foreign pathogens (Duan et al., 2022). Initially discovered in Drosophila melanogaster, Toll was originally identified as a gene involved in establishing dorsal–ventral orientation during embryonic development (Nüsslein-Volhard, 2022). Subsequent research revealed its critical role in Drosophila's immunity to fungal infections (Keith, 2023).

To date, 13 TLRs have been identified in mammals (Liu et al., 2022). TLR1–9 are expressed in both mice and humans, while TLR10–13 are expressed only in mice. Notably, TLR10 is not functional in mice due to a retroviral insertion (Knez et al., 2022). Previous studies have demonstrated that TLR4 mutant mice exhibit a shorter incubation time than control mice in prion disease. However, they did not show significant differences in prion levels (Li et al., 2021). This suggests an involvement of TLR4 in the disease progression. The injection of CpG oligodeoxynucleotides (ODNs), an agonist of TLR9, has been shown to provide protection against prions when administered intraperitoneally (McWhirter and Jefferies, 2022). This finding suggests that innate immune activation interferes with prion infection. In the central nervous system (CNS), innate immunity primarily relies on the functions of glial cells, particularly astrocytes and microglia. These cells play crucial roles in early pathogen control, direct recruitment, and activation of the adaptive immune system necessary for pathogen recognition and clearance (Sanmarco et al., 2021).

*In vivo* studies, TLRs1–9 have been detected in the central nervous system (CNS) using quantitative real-time PCR (Kagoya et al., 2022). Notably, TLR expression levels in the CNS can be up-regulated in response to viral and bacterial infections, exposure to TLR stimuli, or during CNS autoimmunity (Jafarzadeh et al., 2019). This up-regulation provides a mechanism for amplifying inflammatory responses to pathogens that invade the CNS. Microglia, similar to other macrophage-like cells, express virtually all members of the TLR family. In a healthy CNS, TLR expression is minimally detectable in resting microglia. However, upon activation, multiple TLRs rapidly emerge, contributing to the immune response and modulation of microglial function (Fiebich et al., 2018). Primary murine astrocytes exhibit a diverse array of TLRs, albeit at lower expression levels compared to microglia. This observation implies that astrocytes could play a significant role in antiviral responses within the CNS (Telikani et al., 2022). Growing evidence implicates TLRs in neurodegenerative diseases. In both human brains and animal models of Alzheimer's disease, there is an upregulation of TLR2, TLR4, and CD14 expression (Calvo-Rodriguez et al., 2020). Additionally, plaque-associated microglia show elevated mRNA levels for TLR2, TLR4, TLR5, TLR7, and TLR9 (Tan et al., 2021). While innate immune responses have been implicated in the pathogenesis of neurodegenerative diseases, including Alzheimer's disease and transmissible spongiform encephalopathies (Pal et al., 2022), specific reports on TLR expression in prion disease are notably absent (Kim et al., 2020).

In this study, we investigated the features of the 22L prion strain in BALB/C mice. Using qPCR, we measured the expression of TLRs1–13 in the most severely affected region of the CNS. Notably, the cerebellum emerged as the most severely affected disease region in 22L-infected mice. We observed a time-dependent increase in mRNA levels of TLR1, TLR2, TLR7, TLR8, and TLR9 in the cerebellum, mirroring the expression patterns of *PrP*^*Sc*^ (the pathological isoform of the prion protein) and glial fibrillary acidic protein (GFAP) (a marker for gliosis). Remarkably, all TLRs reached elevated levels during the final stages of prion disease. Protein level of TLR7 and TLR9 showed increasing at the end point of the 22L-infected mice. This enhanced understanding of TLR upregulation in prion disease may shed light on their role in initiating immune responses at different stages of pathogenesis, potentially identifying them as therapeutic targets.

## 2 Materials and methods

### 2.1 Murine model of infection

Forty female BALB/C mice, aged 4–6 weeks and weighing 18–22 g, were sourced from the Experimental Animal Center of Jilin University. These mice were intracerebrally inoculated with 20 μl of a 1% brain homogenate (in Sodium Chloride, w/v) derived from a 22L-infected brain obtained from a clinically-infected BALB/C mouse. As controls, mice were also inoculated with 20 μl of a 1% normal brain homogenate. All mice were housed in controlled conditions: 12-h light/dark cycles, a temperature range of 21—22°C, and humidity maintained at 60%–65%. They were individually housed in ventilated cages within a barrier facility and provided *ad libitum* access to food and water. Daily monitoring included observation for neurological signs such as ataxia, muscle tremors, head pressing, hindlimb weakness, paresis, or paralysis. The experimental procedures and animal care protocols were approved by the Committee on Ethical Use of Animals at Jilin University.

### 2.2 Tissue collection

Ten scrapie-inoculated and 10 control animals were humanely sacrificed by cervical dislocation at 60, 90, and 120 days post inoculation (dpi), as well as at the end point (145 dpi). We collected cerebellums and cerebrums from the mice. Half of each brain was frozen directly in liquid nitrogen and stored at −80°C until use. The remaining cerebellum was immediately fixed in 10% formalin for histopathological processing.

### 2.3 Histopathological analysis

The fixed tissues were trimmed, postfixed, and embedded following standard procedures. Tissue sections, 5 μm thick, were cut using a microtome and mounted on treated glass slides (Maixin Company, Fujian, China). Afterward, they were dried overnight at 60°C for 1 h. Finally, the sections underwent routine staining with hematoxylin and eosin.

### 2.4 Immunohistochemical detection of *PrP*^*Sc*^

For the detection of *PrP*^*Sc*^ by immunohistochemistry, histological sections were deparaffinized in xylene, rehydrated in graded alcohols, and washed in distilled water. The following treatments were then carried out to unmask antigens: (1) immersion in 98% formic acid for 15 min; (2) hydrated autoclaving in citrate buffer for 30 min at 121°C. After antigen retrieval, the following steps were included, with intermittent washes using 0.01 M PBS. To inactivate endogenous peroxidase, a solution of 3% hydrogen peroxide in methanol was applied for 15 min, followed by incubation with 5% normal goat serum for 30 min. The primary monoclonal antibody 3H4 (stored in our laboratory) was applied at a dilution of 1:500 and incubated overnight at 4°C (McBride et al., 1988; Demart et al., 1999; Supattapone et al., 2001; Kostelanska and Holada, 2022). Subsequently, sections were treated with biotinylated goat anti-mouse IgG (Maixin, China) for 20 min, followed by streptavidin–horseradish peroxidase (Maixin, China) for 20 min at room temperature. Detection was achieved using diaminobenzidine (Maixin, China) in distilled water for 10 min. Finally, the sections were washed in distilled water and counterstained with Mayer's hematoxylin for 5 min.

### 2.5 Immunohistochemical detection of glial fibrillary acidic protein

Tissue sections were deparaffinized in xylene and rehydrated through graded alcohols. Following antigen retrieval via hydrated autoclaving in citrate buffer for 30 min at 121°C, the sections were sequentially treated as follows:

* Incubation with 3% hydrogen peroxide in methanol at room temperature for 15 min to block endogenous peroxidase;
* Incubation with 5% normal goat serum for 30 min;
* Application of monoclonal mouse anti-GFAP (1:500, BD, USA) at 4°C overnight;
* Use of biotinylated goat antimouse IgG (Maixin, China) as a link antibody for the demonstration of mouse anti-GFAP;
* Application of the streptavidin–biotin–peroxidase (Maixin, China) complex for 20 min;
* Detection using diaminobenzidine for 10 min;
* Sections were then washed in distilled water and counterstained with Mayer's hematoxylin for 5 min.

### 2.6 Western bloting analysis

Cerebellar tissues were homogenized in a lysis buffer containing 50 mM Tris (pH 7.4), 150 mM NaCl, 10 mM EDTA, 0.5% Nonidet P-40, and 0.5% sodium deoxycholate, along with a protease inhibitor cocktail from Roche (Germany). After centrifugation at 12,000 g and 4°C for 30 min, the supernatant was collected and distributed into separate tubes. The final protein concentration was determined using the BCA kit (Beyotime, China). Proteins were mixed with 5 × SDS-PAGE loading buffer (containing 250 mM Tris-HCl, pH 6.8, 50% glycerol, 10% SDS, 0.5% bromphenol blue, and 5% β-mercaptoethanol) and boiled for 5 min. Fifty micrograms of protein were loaded into the sample wells. Protein extracts were electrophoretically separated on a 12% SDS-PAGE gel, run at 120 V for 120 min. The proteins were then transferred to 0.2 μm pore diameter polyvinylidene fluoride (PVDF) membranes (Millipore, USA) using a Semi-Dry Electrophoretic Transfer Cell (Bio-rad, USA) at 15 V for 20 min. The membranes were blocked for 1 h in 5% TBST (0.1% Tween-20 in TBS)–milk and incubated overnight at 4°C with different primary antibodies separately:the primary anti-SAF antibody (Cayman, America) at a dilution of 1:5,000; the primary antibodies of TLR1, TLR2, TLR7, TLR9 (Santa Cruz, USA) at a 1:500 dilution and TLR8 antibody (Santa Cruz, USA) at a 1:200 dilution. After four washes in 0.1% Tween-20 in TBS, the membranes were incubated with horseradish peroxidase-conjugated anti-mouse antibody (Santa Cruz, USA) at a 1:5,000 dilution for 1 h at room temperature, separately. Immune complexes were detected using an electrochemiluminescence (ECL) kit (Amersham, USA) on Hyperfilm MP (Amersham, USA). ECL Plus Western Blotting Substrate (Applygen, China) was used according to the supplier's instructions. In *PrP*^*sc*^ detction, Cerebellar tissues were made as mentioned above. The samples in both the control group and the 22L-infected group were digested with PK (Sigma, USA) at the temperature of 37°C for 1h. Fifty micrograms of protein were loaded into the sample wells. The primary anti-SAF antibody (Cayman, America) at a dilution of 1:5,000.

### 2.7 RNA extraction and reverse transcription

Total RNA was extracted from the cerebellum of five 22L-infected and five mock-infected BALB/C mice at 60, 90, 120 dpi, and at the end point (145 dpi) using TRIzol (Invitrogen, USA) according to the manufacturer's instructions. Reverse transcription polymerase chain reaction (RT-PCR) was performed using Reverse Transcriptase M-MLV (RNase *H*^−^) (Takara, China). One microgram of total RNA was reverse transcribed to cDNA in a 20 μl reaction solution following the manufacturer's instructions (Takara, China). The reaction was carried out at 30°C for 5 min, 42°C for 60 min, and 70°C for 15 min. The resulting cDNA was stored at −20°C until use in downstream Quantitative PCR.

### 2.8 Quantitative PCR

Quantitative PCR was performed to analyze the mRNA expression levels of all mouse TLRs (TLR1-13) and GFAP using the Applied Biosystems 7300 Real-Time PCR System (Applied Biosystems, USA). To account for variations in mRNA amounts, GAPDH was included as a housekeeping gene. Each 25 μl reaction system contained 12.5 μl 2x SYBR Green PCR Master Mix (Applied Biosystems, USA), 2.5 μl cDNA (10 ng/μl), and 5 μl of each primer (2.5 μM). The primer sequences for each gene are presented in Table 1. Reactions were carried out under qPCR conditions, including denaturation at 95°C for 10 min, followed by 40 cycles of amplification (95°C for 15 s, 60°C for 1 min). Subsequently, a melting curve analysis and the ΔΔCt method were used for data processing.

**Table 1:** Primers of mouse TLRs (mTLRs), GFAP and GAPDH.

**Gene**	**Sense primer (5^′^ → 3^′^)**	**Amplicon size**
mTLR1	5′-CTGAGGGTCCTGATAATGTCCTAC-3′	114 bp
	5′-GATCACCTTTAGCTCATTGTGGG-3′	
mTLR2	5′-TTGCGTTACATCTTGGAACTG-3′	92 bp
	5′-ACTACGTCTGACTCCGAGGG-3′	
mTLR3	5′-CAACGGTTCCTTCTCCTATCTC-3′	139 bp
	5′-TTGCTTAGTAAATGCTCGCTTC-3′	
mTLR4	5′-CTTCATTCAAGACCAAGCCTTTC-3′	126 bp
	5′-AACCGATGGACGTGTAAACCAG-3′	
mTLR5	5′-TCTACAACATATCCACCGAAGACTG-3′	123 bp
	5′-TTATGACTACAAGGGTGATGACGAG-3′	
mTLR6	5′-GTACCGTCAGTGCTGGAAATAGAG-3′	92 bp
	5′-GCTCATGTTGCAGAGGCTATCC-3′	
mTLR7	5′-TGAGGGCATTCCCACTAACAC-3′	96 bp
	5′-TCCAGATGGTTCAGCCTACGG-3′	
mTLR8	5′-TTCCTCACATTCCTTACCACCTC-3′	87 bp
	5′-GTGATAGATAAACCAAACATCCCAG-3′	
mTLR9	5′-TCTGTCTTACTACACCGCTATTTG-3′	93 bp
	5′-AAACTACCCTTTACAGCCAACC-3′	
mTLR11	5′-ATAGGCAGAGGCTCCATAGTTAC-3′	107 bp
	5′-TTGCTCACAGAAAGAGTTCCAC-3′	
mTLR12	5′-GGTCTCCCGCTATTTCACATTC-3′	108 bp
	5′-ACAGTCCGAGGTACAACTTCCA-3′	
mTLR13	5′-CCTCTGTTGCATGATGTCGAG-3′	197 bp
	5′-CTCCCATTCATCTTGACTGTCTT-3′	
GFAP	5′-GAGAACAACCTGGCTGCGTATAGAC-3′	93 bp
	5′-CTCCTCCTCCAGCGATTCAACCT-3′	
GAPDH	5′-GACTTCAACAGCAACTCCCACTC-3′	107 bp
	5′-TAGCCGTATTCATTGTCATACCAG-3′	

### 2.9 Statistical analysis

All data are expressed as the mean value ± standard deviation (SD), and each resulting value was determined by averaging three independent experiments. Western blot data and qPCR results were analyzed using one-way ANOVA, followed by Tukey's multiple range tests in SPSS 14.0. Differences were considered statistically significant at *P* < 0.05.

## 3 Results

### 3.1 Clinical signs, spongiform changes, gliosis and *PrP*^*Sc*^ deposition

To analyze the features of the 22L strain in BALB/C mice, we observed clinical signs, spongiform changes, and *PrP*^*Sc*^ deposition. All 22L-infected mice exhibited clear scrapie-associated clinical signs at ~120 days post-infection (dpi). These signs included rough coat, weight loss, and abnormalities in motor function such as tremors, ataxia, and late-stage recumbency. Changes in mental status were also frequently observed, with hyperexcitability at the early onset and depression at the end point. In our research, we cultivated several groups of mice. The first group was utilized to measure the duration from 22L inoculation to the natural death of the mice. At the time from inoculation to the final death was 142.6 ± 5.0 days. The incubation period defined as the time between the initial exposure to prions and the first appearance of symptoms. This experiment yielded an average incubation period of 120.7 ± 4.5 days. For subsequent groups of mice, we delineated four stages of pathogenesis based on this average incubation period: 60, 90, 120, and 145 dpi. Notably, the 145 dpi stage signifies the disease's end point.

**Figure 1:**
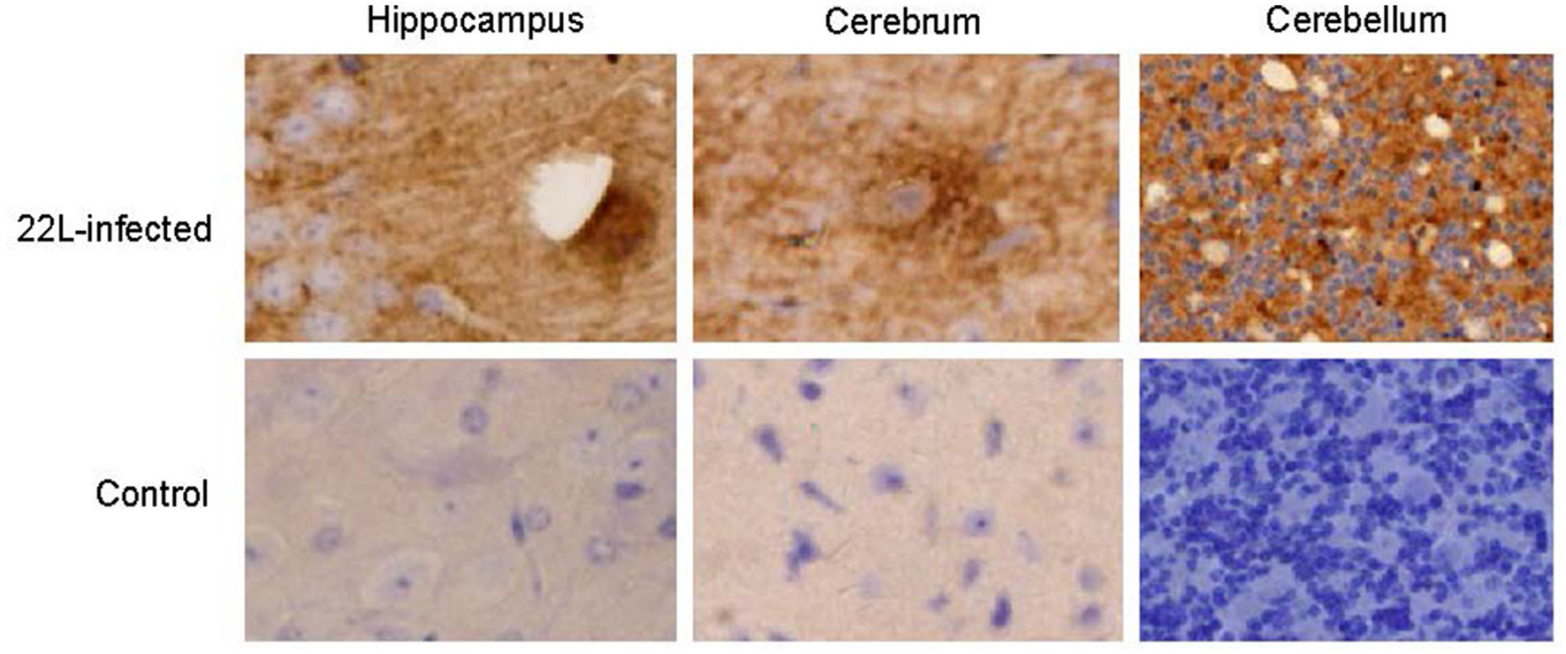
Coronal sections of the hippocampus, cerebrum, and cerebellum from 22L-infected at the end point and the control mice reveal distinct *PrP*^*Sc*^ deposition patterns. These sections were immunoassayed with the monoclonal 3H4 antibody and photographed at a magnification of × 400. Dark brown areas indicate the positions of *PrP*^*Sc*^ accumulation. Notably, the style of *PrP*^*Sc*^ deposition differed in 22L-infected BALB/C mice. Specifically: Plaque-like *PrP*^*Sc*^ deposits were observed in the cortex and hippocampus. Diffuse *PrP*^*Sc*^ deposits were present in the cerebellum. In contrast, control sections showed an absence of *PrP*^*Sc*^ detection.

**Figure 2:**
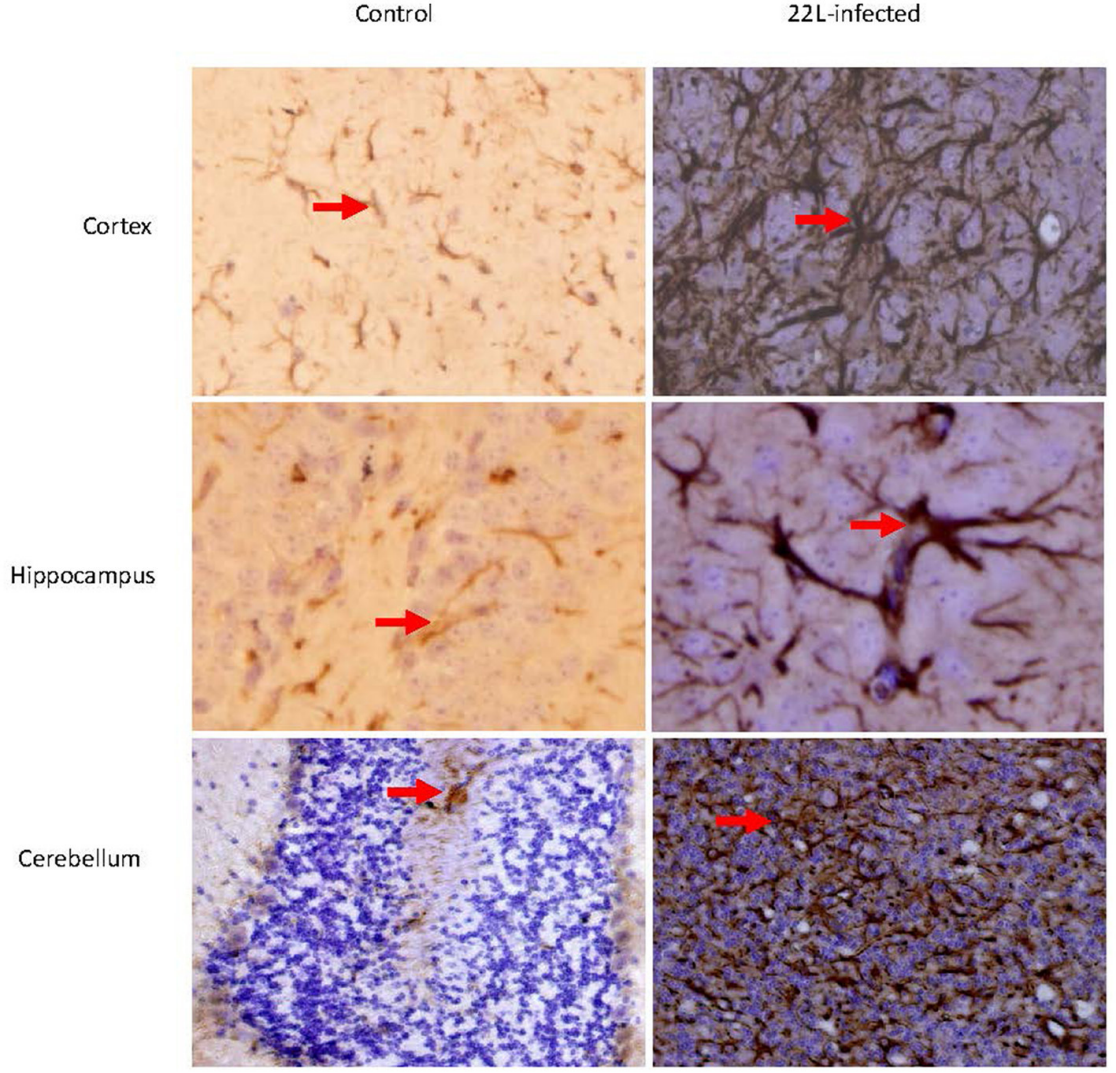
Coronal sections of the Cortex, Hippocampus, and cerebellum from 22L-infected at the end point and control mice revealed distinct GFAP expression. These sections were immunoassayed with the monoclonal GFAP antibody and photographed at magnification of × 400. Dark brown areas indicated the positions of GFAP expression (arrow pointing). Notably, GFAP expression increased in in the brains of scrapie-affected mice, with the cerebellum showing the most intense staining.

Histological examination using HE staining revealed widespread vacuolation in both white matter and gray matter of the brain, particularly in the cerebrum, cerebellum, and spinal cord. The cerebellum exhibited a higher level of vacuolation, especially in granulosa cells and Purkinje's cells (data not shown). Immunohistochemistry was used to measure *PrP*^*Sc*^ deposition and the expression of glial fibrillary acidic protein (GFAP), an astroglial marker. The most intense deposition of *PrP*^*Sc*^ was observed within the hippocampus, cerebrum, and cerebellum. The defining characteristic of scrapie is the vacuolation of neurons, gliosis, and *PrP*^*Sc*^ deposits. Different strains of scrapie may exhibit unique features. Through immunohistochemistry, we observed that *PrP*^*Sc*^ was deposited in various parts of the CNS in 22L-infected mice, including the cortex, hippocampus, cerebrum, cerebellum, and spinal cord. However, the expression of *PrP*^*Sc*^ varied across these areas. In Table 2, we evaluated the vacuolation, gliosis, and *PrP*^*Sc*^ deposits in 22L-infected mice. Our findings revealed that the cerebellum was the most intensely affected region in 22L-infected BALB/C mice. Specifically, the 22L strain showed plaque-like *PrP*^*Sc*^ deposits in the cortex and hippocampus, while diffuse *PrP*^*Sc*^ deposits were seen in the cerebellum (Figure 1). Additionally, there was an increase in GFAP expression in the brains of scrapie-affected mice, with the cerebrum and cerebellum showing the most intense staining (Figure 2). The deposition of *PrP*^*Sc*^ and GFAP correlated with the severity of spongiosis and vacuolation. Spongiform changes, *PrP*^*Sc*^ deposition, and gliosis were scored (García-Martínez et al., 2022) (Table 2). The cerebellum, as the most intensely affected region in 22L-infected BALB/C mice, was selected for further study.

**Table 2:** Neuropathology in BALB/C mice infected with 22L strain.

**Brain's anatomy**	**Vacuolation**	**Gliosis**	***PrP*^*Sc*^ accumulation**
Cortex	+++	+++	+++
Hippocampus	+	+	++
Brainstem	+++	++	+++
Cerebellum	+++	+++	++++
Spinal cord	++	+	++

### 3.2 Changed TLRs expression during the pathogenesis of prion disease

**Figure 3:**
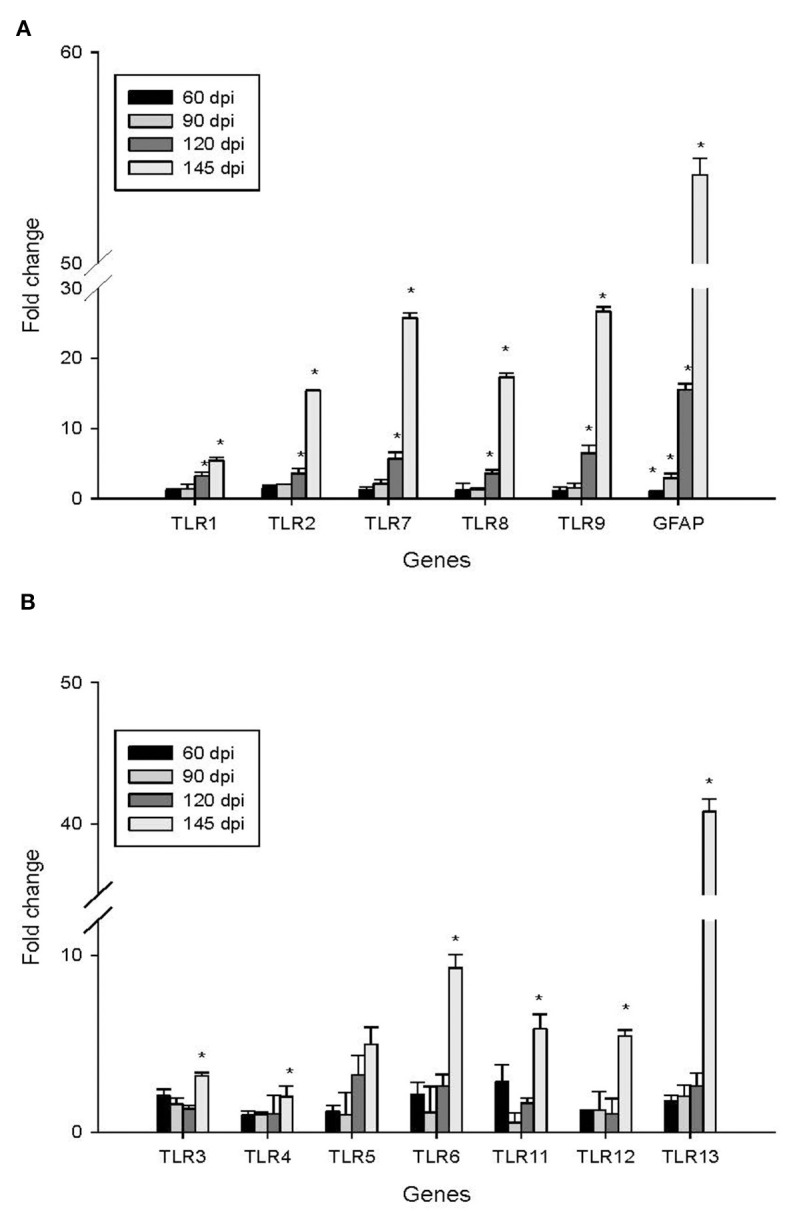
Changes in the expression of TLR1-13 and GFAP in the cerebellum during the pathogenesis of prion disease were investigated at different time points (60, 90, 120, and 145 days post-infection, dpi). The results are summarized as follows: Subgraph **(A)**: Levels of TLR1, TLR2, TLR7, TLR8, TLR9, and GFAP. Subgraph **(B)**: Expression of TLR3, TLR4, TLR5, TLR6, TLR11, TLR12, and TLR13. The mRNA expression levels were quantified using quantitative real-time PCR analysis. The values (fold change) represent the proportion of expression levels of TLRs and GFAP in 22L-infected mice compared to those in control mice (mean ± standard deviation, *n* = 4). An asterisk (*) indicates that the expression of a specific gene was statistically significant (*P* < 0.05) when compared with the expressions at any dpi.

**Figure 4:**
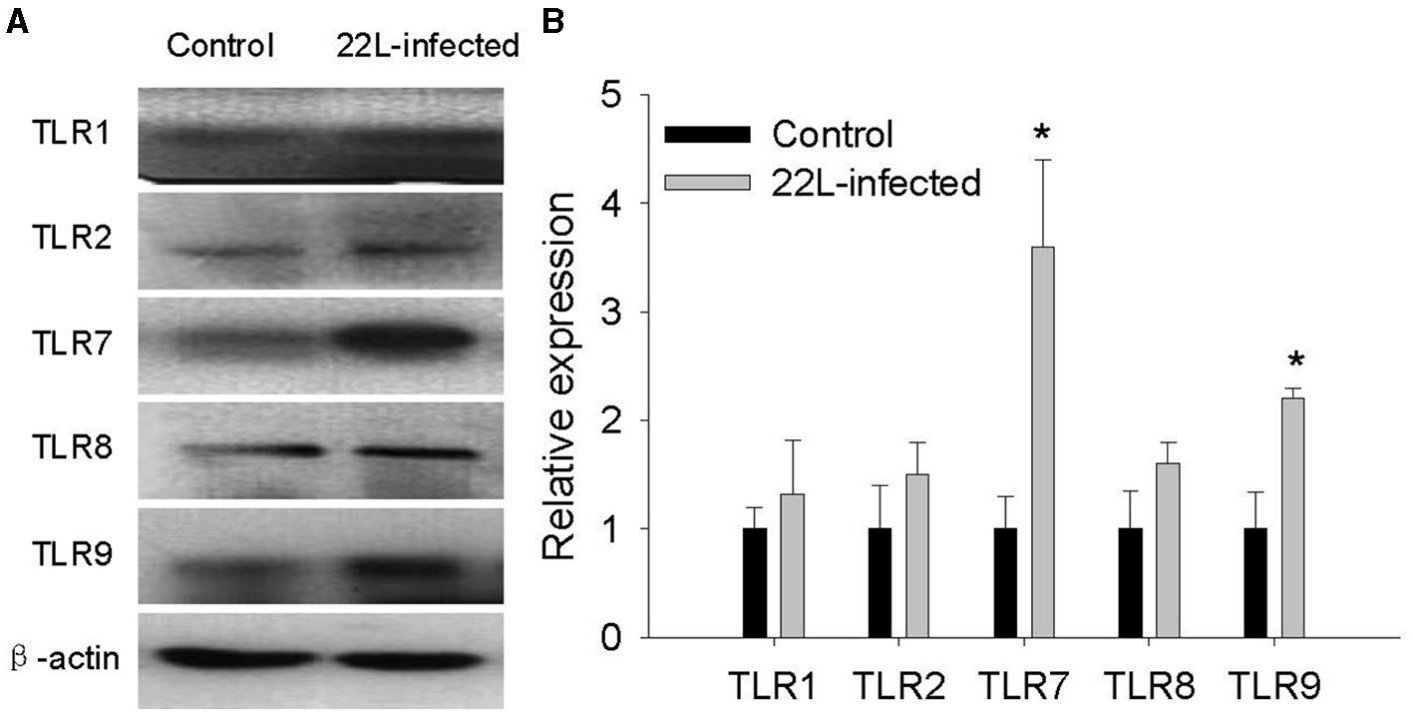
Detection of TLR1, TLR2, TLR7, TLR8, and TLR9 expression in the cerebellum of 22L-infected mice at the end point and controls, as revealed by Western Blotting. **(A)** The proteins were detected using antibodies specific to TLR1, TLR2, TLR7, TLR8 and TLR9, respectively. β-actin, was used as a control and detected with a corresponding antibody. **(B)** The histogram shows the expression levels of TLR1, TLR2, TLR7, TLR8 and TLR9, calculated by grayscale value relative to β-actin. The values are represented as the fold change in comparison to the Control mice, with normalization to 1, ^*^P < 0.05. The experimental data reveal statistical significance for TLR7 and TLR9.

To investigate the impact of the 22L strain on Toll-like receptor (TLR) expression during pathogenesis, we measured the expression levels of mouse TLRs (TLR1-13) using qPCR, along with glial fibrillary acidic protein (GFAP). Cerebellums from 22L-infected (*n* = 4) and control groups (*n* = 4) were collected at 60, 90, 120 dpi, and the end point (145 dpi). Total RNA was extracted, and cDNA was synthesized. Our results revealed that the expressions of TLR1, 2, 7, 8, and 9 increased in a time-dependent manner during pathogenesis. To explore the relationship between TLR levels and glial cells, we also measured GFAP expression at the same time. Interestingly, GFAP expression followed similar trends to these TLRs. However, the levels of TLR3, 4, 5, 6, 11, and 12 exhibited fluctuations before clinical signs appeared (at 120 dpi). Notably, TLR1-13 reached relatively high levels at the final stage (145 dpi) (Figure 3). To detect whether these TLRs expressions increased in protein level, we verified the expression levels of TLR1, -TLR2, -TLR7, -TLR8 and -TLR9 by Western blotting. Cerebellums from 22L-infected (*n* = 4) at the end point, and control groups (*n* = 4) were collected. Total protein was extracted. The proteins were detected using anti-TLR1, -TLR2, -TLR7, -TLR8 and -TLR9 antibodies, respectively. β-actin, was used as a control. The results showed that TLR7 and TLR9 expressions increased at the end point of the 22L-infected mice in protein level (Figure 4).

### 3.3 Relationship of *PrP*^*Sc*^ accumulation, gliosis and TLRs expression

**Figure 5:**
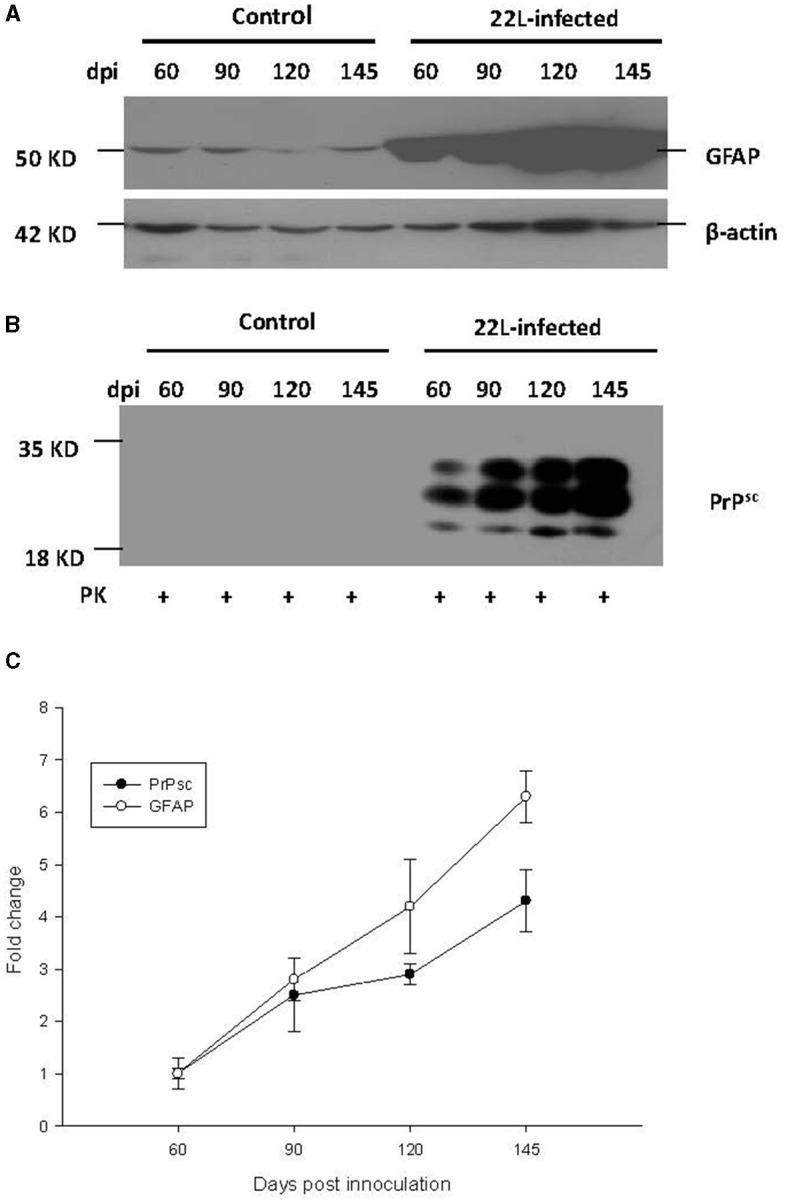
During the pathogenesis of 22L-infected mice, the expressions of *PrP*^*Sc*^ (abnormal isoform of the prion protein) and GFAP (glial fibrillary acidic protein) in the cerebellum increased at 60, 90, 120, and 145 days post-infection (dpi). Total protein was extracted from the cerebellums, and 50 μg of protein was loaded into each lane. Here are the details: **(A)** GFAP Expression: We detected GFAP expression using western blot analysis with a monoclonal mouse anti-GFAP antibody. β-actin served as the housekeeping protein reference. **(B)** *PrP*^*Sc*^ Level: The level of *PrP*^*Sc*^ was measured by western blot using a monoclonal mouse anti-SAF antibody. **(C)** Fold change analysis: we analyzed the fold change of GFAP and *PrP*^*Sc*^ expression across different dpi using SigmaPlot. Notably, both GFAP and *PrP*^*Sc*^ exhibited time-dependent changes during the pathogenesis of prion disease. Please note that these findings provide valuable insights into the molecular events associated with prion infection in the cerebellum.

To investigate the relationship between *PrP*^*Sc*^ accumulation, gliosis, and TLR expression, we examined protein levels of GFAP and *PrP*^*Sc*^ in the cerebellum at 60, 90, 120, and 145 days post-infection (dpi). Our findings revealed the following:

* GFAP expression: the expression of GFAP increased in a time-dependent manner, consistent with the progression of the disease (Figure 5).
* *PrP*^*Sc*^ accumulation: similarly, the levels of *PrP*^*Sc*^ also showed a time-dependent increase, mirroring the pattern observed for GFAP and TLRs mentioned earlier (Figure 5). In both the control group and the 22L-infected group, the samples were digested with PK at a temperature of 37 °C for 1 h, and precisely 50 μg of protein was loaded into each lane.
* Final stage: notably, at the final stage (145 dpi), both GFAP and *PrP*^*Sc*^ reached relatively high levels, suggesting their involvement in the pathogenesis of prion disease.

These results provide valuable insights into the interplay between glial activation, abnormal prion protein accumulation, and immune responses during prion infection.

## 4 Discussion

CNS is often considered an immune-privileged site due to its unique characteristics, including the absence of a classically defined lymphatic system (González-Hernández and Mukouyama, 2023). Despite this privilege, innate immunity likely plays a crucial role in controlling pathogen invasion within the CNS. Pattern recognition receptors (PRRs), which recognize pathogen-associated molecular patterns (PAMPs), may contribute to altered brain homeostasis and various CNS diseases (Chen et al., 2017). These diseases encompass a wide range of causes, including experimental brain injuries resulting from stereotactic transection of axons in the entorhinal cortex, ischemic events, and autoimmune conditions.

Previous studies have demonstrated that the Toll-like receptor (TLR) family plays a critical role in host innate immunity (Sameer and Nissar, 2021). Researchers have extensively analyzed the expression of TLRs in the CNS of rodents and humans. Moreover, growing evidence suggests that TLRs are implicated in neurodegenerative diseases. Notably:

* Alzheimer's disease (AD): increased expression of TLR2, TLR4, and CD14 has been observed in human brains and animal models of AD (Su et al., 2016). Additionally, plaque-associated microglia exhibit elevated mRNA levels for TLR2, 4, 5, 7, and 9.
* Prion diseases: surprisingly, despite the importance of innate immune responses in neurodegenerative pathogenesis (Labzin et al., 2018), there have been no reports specifically addressing the expression patterns of TLRs during prion diseases (Zhang et al., 2020).

Understanding the interplay between TLRs and neurodegenerative processes remains an intriguing area of research, and further investigations into TLR expression dynamics during prion disease could provide valuable insights.

In this study, a mouse model of prion disease (specifically the 22L strain) was established. After identifying the distinctive features of the 22L strain, researchers selected the most severely affected region. Subsequently, they analyzed the expression levels of TLRs in this region. Notably, this report represents the first description of prion-related alterations in these innate immune receptors within the cerebellum during the pathogenesis of prion disease.

Various distinct prion strains have been identified in mice through serial passages of scrapie, BSE, or CJD originating from sheep, goats, cattle, or humans. Researchers utilized inbred mouse strains to establish prion-infected mouse models, aiming to investigate the mechanisms underlying prion diseases due to their shared clinical symptoms and associated pathological changes. The inoculation of scrapie into inbred mouse strains, along with consideration of the host's genetic background, plays a crucial role in determining the neuropathological lesions and incubation periods associated with prion diseases. In our study, we investigated the features of the 22L strain in female BALB/C background mice. Specifically, we examined the position of vacuolation and the pattern of *PrP*^*Sc*^ accumulation using HE staining and immunohistochemistry. Our data revealed widespread distribution of vacuoles throughout the brain, particularly in the cortex and cerebellum. Notably, the vacuolation score indicated severe spongiform changes in the cerebellum. Additionally, 22L strain infection in BALB/C mice was characterized by plaque-like *PrP*^*Sc*^ deposits in the cortex and hippocampus, along with diffuse *PrP*^*Sc*^ deposition in the granular layer of the cerebellum, suggesting elevated *PrP*^*Sc*^ expression in this region. Consequently, we conclude that the cerebellum represents the most severely affected disease region in BALB/C mice infected with the 22L strain. We quantified the expressions of TLR1-13 in the cerebellum during the pathogenesis of 22L strain-infected mice and control mice at various time points (60, 90, 120, and 145 days post-infection). Our findings demonstrate that mRNA for all tested TLRs was detectable in both 22L strain-infected mice and control mice. Notably, the levels of TLR1, 2, 7, 8, and 9 exhibited a time-dependent increase during the course of pathogenesis. By Western blotting, expressions of TLR7 and TLR9 in protein level showed increasing at the end point of 22L strain-infected mice to the control mice.

During the late stages of prion disease, the expressions of TLR1-13 significantly increased. However, TLR3, TLR4, TLR6, TLR12, and TLR13 exhibited less pronounced changes in expression prior to reaching the end point. Additionally, GFAP expression was also measured and demonstrated a time-dependent pattern. Expression of GFAP in cerebrum and cerebellum of 22L-affected mice, were measured by immunohistochemistry, as shown in Figure 2. By considering the similarity in amino acid composition, extracellular leucine-rich repeat (LLR) length, and phylogenetic analysis, researchers identified six subfamilies. Specifically, the TLR2 subfamily comprises TLR1, TLR2, and TLR6; the TLR9 subfamily includes TLR7, TLR8, and TLR9, and the TLR11 subfamily encompasses TLR11, TLR12, and TLR13. Furthermore, the TLR3, TLR4, and TLR5 subfamilies consist of a single member each. Our data revealed that the TLR2 and TLR9 subfamilies exhibited increased expression during the pathogenesis of prion disease. At the end point, TLR7 and TLR9 expression in protein level increased. Previous studies have reported that TLR7 increased in the thalamus in FFI (Llorens et al., 2016), and it mean inflammation might take part in pathogenesis of scrapie. A study reported an upregulation of TLR2 expression in 22L C57BL/6 infected mice. Interestingly, TLR2 knockout mice exhibited accelerated disease progression, with a median reduction of 10 days. These findings suggest that TLR2 signaling plays a partially protective role during prion infection (Carroll et al., 2018). It was reported that astrocytes express low levels of mRNA for TLR2, TLR4, TLR5, and TLR9, with further elevation upon activation by specific TLR ligands. Additionally, resting astrocytes express moderate levels of TLR1 and TLR6, along with very low levels of TLR7 and TLR8. Notably, TLR2 ligand recognition in the peripheral immune system typically involves TLR2 dimerization with either TLR1 or TLR6. The similarity in the expression patterns of TLR1 and TLR2 (but not TLR6) in the cerebellum suggests a functional association between TLR1 and TLR2 in this brain region.

During prion disease, *PrP*^*Sc*^ accumulation in the CNS leads to gliosis, primarily involving astrocytes. Remarkably, the mRNA expression of TLR1, TLR2, TLR7, TLR8, TLR9, and GFAP followed a consistent pattern, peaking at the final disease stage. Protein level of TLR7 and TLR9 showed increasing at the end point of the mice. This observation suggests that astrocytes may actively contribute to the elevated expression of TLRs, especially considering the concurrent loss of neurons and the reactive proliferation of glial cells. To further investigate, we quantified protein levels of *PrP*^*Sc*^ and GFAP in the cerebellum via western blot analysis. Our findings revealed that both *PrP*^*Sc*^ and GFAP increased progressively over time. This dynamic pattern suggests that *PrP*^*Sc*^ accumulation and glial cell proliferation escalate during the course of prion disease. Within the CNS, neurons and glial cells are the primary cell types expressing TLRs. Firstly, *PrP*^*Sc*^ accumulation may trigger neuron loss and enhance glial cell proliferation. Consequently, the expanded glial cell population likely contributes to the elevated TLR expression. Secondly, *PrP*^*Sc*^ itself could directly modulate TLR levels in the CNS as a pathogenic factor. Notably, accumulating evidence underscores the central role of TLRs in diverse inflammatory CNS pathologies. In the early stages of prion disease, the increased TLR levels and associated immune responses may assist the host in pathogen clearance. However, during the final disease stage, the sharp elevation in TLR expression could lead to severe immune responses detrimental to the CNS, particularly affecting neurons. Notably, previous studies have linked TLR9 expression to prion disease progression, as evidenced by responses to synthetic oligodeoxynucleotides. Furthermore, TLR4 mutant mice exhibited a shorter incubation time than control mice in prion disease. When considering TLRs as potential therapeutic targets for prion disease, immune responses that correlate with TLR levels during pathogenesis merit careful consideration.

## 5 Conclusion

In our current study, we demonstrated the expression of TLR1-13 in the cerebellum, which represents the severely affected region within the CNS. Specifically, the expression of TLR1, TLR2, TLR7, TLR8, and TLR9 exhibited significant upregulation in a time-dependent manner during the course of pathogenesis. Notably, all TLRs (TLR1-13) reached elevated levels at the final stage of prion disease. Protein level of TLR7 and TLR9 showed increasing at the end point of the 22L-infected mice. Additionally, both mRNA and protein levels of GFAP, along with protein expression of *PrP*^*Sc*^, increased progressively over time during the pathogenesis of prion disease. A deeper understanding of the heightened TLR expression in prion disease may offer valuable insights into their involvement in initiating immune responses at various stages of pathogenesis. Importantly, this understanding could inform the exploration of TLRs as potential therapeutic targets for prion disease.

## Data availability statement

The datasets presented in this study can be found in online repositories. The names of the repository/repositories and accession number(s) can be found in the article/supplementary material.

## Ethics statement

The animal studies were approved by the Committee on Ethical Use of Animals at Jilin University. The studies were conducted in accordance with the local legislation and institutional requirements. Written informed consent was obtained from the owners for the participation of their animals in this study.

## Author contributions

XiaL: Conceptualization, Data curation, Methodology, Project administration, Resources, Supervision, Validation, Writing—original draft. WZ: Conceptualization, Data curation, Formal analysis, Methodology, Resources, Validation, Visualization, Writing—review & editing. XinL: Data curation, Writing—review & editing, Visualization, Validation, Software, Methodology, Funding acquisition, Formal analysis. WL: Conceptualization, Data curation, Formal analysis, Resources, Validation, Writing—review & editing. YH: Validation, Writing—review & editing. JW: Conceptualization, Data curation, Formal analysis, Resources, Supervision, Validation, Writing—review & editing.
